# Antioxidant, anti-inflammatory, and phytochemical properties of *Juniperus osteosperma* (Torr.) little hydrosol: an *in vitro* evaluation

**DOI:** 10.3389/fphar.2026.1774954

**Published:** 2026-06-24

**Authors:** Chris Packer, Valeria Calero-Torres, Adrian Abad, Liz Burgos-Moreira, Marcela Muzzio, Juan Manuel Cevallos, Patricia Manzano-Santana, Andrea Orellana-Manzano

**Affiliations:** 1 D. Gary Young Research Institute, Lehi, UT, United States; 2 Laboratorio de Farmacología Molecular Aplicada, Facultad de Ciencias de la Vida, Escuela Superior Politécnica del Litoral, ESPOL, Campus Gustavo Galindo, Guayaquil, Ecuador; 3 Facultad de Ciencias de la Vida, Escuela Superior Politécnica del Litoral (ESPOL), Guayaquil, Ecuador; 4 Centro de Investigaciones Biotecnológicas del Ecuador, Escuela Superior Politécnica del Litoral (ESPOL), Campus Gustavo Galindo, Guayaquil, Ecuador

**Keywords:** anti-inflammatory, antioxidant, COX- cyclo-oxygenase, hydrosol, Juniperus osteosperma (Torr.) little, microbiology

## Abstract

The hydrosol of *Juniperus osteosperma* (Torr.) Little has scarcely been characterized from a chemical and biological perspective. In this study, the phytochemical profile and *in vitro* antioxidant, anti-inflammatory, and antibacterial activities of the plant were evaluated. Antioxidant activity was assessed using the FRAP, ABTS, and DPPH assays, while total phenolic and flavonoid contents were determined using the Folin Ciocalteu and aluminum chloride methods, respectively. Anti-inflammatory activity was assessed by inhibiting cyclooxygenase enzymes COX-1 and COX-2, and antibacterial activity was evaluated using the agar diffusion method. HPLC-PDA analysis revealed the tentative detection of naringenin as the only quantifiable flavonoid under the chromatographic conditions employed, consistent with the restricted phytochemical profile typically associated with hydrosols. The hydrosol exhibited antioxidant activity and a moderate, dose-dependent inhibition of COX-1 and COX-2, with the highest effects observed at elevated concentrations, whereas no antibacterial inhibition zones were detected using the applied method, a result that may be attributable to methodological limitations. Overall, these findings contribute to the chemical and biological characterization of *J. osteosperma* (Torr.) Little hydrosol and support the need for future studies that incorporate complementary analytical techniques and cellular and toxicological models to further investigate its bioactive potential.

## 1 Introduction

*Juniperus osteosperma* (Torr.) Little*,* commonly known as Utah juniper, is one of the dominant species of the semi-arid woodlands of the Great Basin (United States of America), with a distribution ranging from New Mexico and Wyoming to the Sierra Nevada and the Mojave Desert (Gonçalves et al., 2022; Wilson et al., 2021). Morphologically, it is a large shrub or small tree reaching 6–9 m in height, characterized by a gray-brown fibrous bark, dark green needle-like leaves, and a dioecious reproductive system, producing bluish-purple galbuli measuring 6–12 mm in diameter. Its high drought tolerance allows it to form dominant plant communities together with Pinus monophylla in xerophytic regions of the southwestern United States (Swor et al., 2024; Vasek, 1966).

Utah juniper plays a key ecological role in arid ecosystems and holds ethnobotanical relevance, as several Native American tribes traditionally used its resin, bark, and leaves to treat rheumatism, respiratory disorders, dermatological conditions, and digestive ailments, attributing purifying and anti-inflammatory properties to the species (Swor et al., 2024).

The genus Juniperus comprises approximately 68 species and has been extensively investigated from a phytochemical perspective. Numerous studies have demonstrated that different plant organs (leaves, berries, bark, and wood) contain a wide variety of bioactive metabolites, including monoterpenes (α-pinene, sabinene, limonene), oxygenated monoterpenoids (borneol, terpinen-4-ol, camphor), sesquiterpenes (cedrol), and phenolic compounds such as flavonoids (quercetin, rutin), biflavonoids (amentoflavone), and phenolic acids (Miceli et al., 2011; Kalam, 2020; Gonçalves et al., 2022). The chemical composition and relative abundance of these compounds depend strongly on the plant organ, extraction method, and matrix type analyzed.

In terms of biological activity, organic extracts and essential oils from different Juniperus species have been reported to exhibit antioxidant, anti-inflammatory, and antimicrobial properties. Gonçalves et al. (2022) reported a wide variability in the antioxidant activity of Juniperus communis extracts, with IC_50_ values against the DPPH radical ranging from 1.42 to 257 μg/mL, depending on the solvent employed. Additional studies have demonstrated anti-inflammatory activity through the inhibition of key enzymes, such as cyclooxygenase-2 (COX-2) and 5-lipoxygenase, particularly in ethanolic extracts rich in terpenes and lipophilic polyphenols (Zhao et al., 2018; Gonçalves et al., 2022). Regarding antimicrobial activity, Karaman et al. (2003) evaluated aqueous and methanolic leaf extracts of *Juniperus oxycedrus* against a broad range of bacteria and fungi, observing that aqueous extracts lacked activity, whereas methanolic extracts inhibited the growth of 57 strains across several genera. These findings indicate that antimicrobial activity within the genus Juniperus is highly dependent on extract type, solvent polarity, and the microorganism evaluated.

Hydrosols, obtained as aqueous by-products of steam distillation of essential oils, represent a distinct chemical matrix characterized by the presence of water-soluble volatile compounds at low concentrations. In Juniperus species, hydrosols have been described as enriched in relatively polar oxygenated monoterpenoids, such as terpinen-4-ol and borneol, in contrast to essential oils, which are typically dominated by hydrocarbon monoterpenes (Fernandez and Cock, 2016; Semerdjieva et al., 2021). Due to their aqueous nature and the low solubility of non-volatile compounds, hydrosols are generally expected to contain a more restricted phytochemical profile compared to organic extracts.

To date, no previous studies have systematically characterized the chemical composition or bioactive profile of *J. osteosperma* (Torr.) Little hydrosol. In this context, the present study aims to characterize the chemical profile of *J. osteosperma* (Torr.) Little essential oil and to evaluate the antioxidant, anti-inflammatory, and antibacterial activities of the hydrosol obtained as a by-product of its distillation, thereby contributing to the scientific understanding of this species.

## 2 Methodology

### 2.1 Plant material collection

In the present study, the starting material was the secondary product (hydrosol) obtained during the steam distillation of the essential oil of *J. osteosperma* (Torr.) Little (Utah juniper) produced by Young Living. The plant material was collected from a native population located in the central-western region of the state of Utah, United States. The sampling site is a semi-arid rural area located at 39.7901431° N, 111.9601310° W. A representative voucher specimen was deposited in the Young Living Aromatic Herbarium (YLAH) as *J. osteosperma* (Torr.) Little, Wilson 2019-000079 (YLAH). The essential oil was obtained from trunks, branches, and leaves.

### 2.2 Process of obtaining the hydrosol

The *J. osteosperma* (Torr.) Little hydrosol was obtained by steam distillation, in accordance with Young Living’s internal methodology. The wood material was processed into chips approximately 2″-4″ inches in size using a Vermeer HG4000 Horizontal Grinder. The chips were loaded into a 7,800 L distillation vessel. Steam used in the process was generated by a Clayton steam generator operating at 100 psi and 170 °C. A total of 2,500 kg of *J. osteosperma* (Torr.) Little wood chips were used, and the distillation process was conducted for 4 h under a constant steam flow. The resulting condensate was collected and separated by density into essential oil (upper phase) and hydrosol (lower aqueous phase).

### 2.3 Sample preparation

Antioxidant assays, as well as the determination of total phenolic content (TPC), flavonoid content (TFC), and antimicrobial activity, were performed using *J. osteosperma* (Torr.) Little hydrosol in its native form (100%), considering its aqueous nature and low concentration of bioactive compounds. This allows for the evaluation of its activity under conditions close to its original state and ensures an adequate analytical signal.

In contrast, for the evaluation of anti-inflammatory activity, the same hydrosol was subjected to a pre-concentration process by evaporation under reduced pressure at 40 °C (Vacufuge Plus, 3 h) in order to increase the concentration of bioactive compounds and allow the detection of inhibitory activity on COX-1 and COX-2 enzymes. The resulting residue, corresponding to the concentrated hydrosol, was subsequently resuspended in 1.3% DMSO (20 mg/mL), sonicated for 15 min at 25 °C, and stored at 4 °C until analysis.

### 2.4 Chemical characterization of the essential oil

The chemical characterization of the essential oil of *J. osteosperma* (Torr.) Little was carried out to complement the analysis of the hydrosol. The oil, provided by Young Living (United States) and obtained by steam distillation, was analyzed by GC–MS following a modified methodology of (Wilson et al., 2023). A GC Agilent 7890B system coupled to a 5977B MSD and a DB-5 capillary column (60 m × 0.25 mm, 0.25 µm) was used. A 0.1 µL injection was made in split 25:1. The oven program started at 40 °C (5 min), increasing at 4.5 °C min^-1^ up to 310 °C (5 min). Helium served as the carrier gas at a constant flow rate. The MS was operated with EI at 70 eV, a mass range of 35–650 amu, 2.4 scans s^-1^, the source at 230 °C, and the quadrupole at 150 °C. The compounds were identified using the Adams volatile oil library and a ChemStation library search, in conjunction with retention indices (KI) (Wilson et al., 2023; Wilson et al., 2021; Wilson et al., 2019; Yang et al., 2014; Kim et al., 2015).

### 2.5 Antioxidant activity

#### 2.5.1 DPPH (2,2-diphenyl-1-picrylhydrazyl)-assay

The antioxidant activity of *J. osteosperma* (Torr.) Little hydrosol was evaluated using the DPPH assay (156 µM) as described by Viteri et al. (2022). Briefly, 50 µL of the sample was mixed with 150 µL of DPPH solution in a 96-well microplate, incubated in the dark for 30 min, and the absorbance was measured at 517 nm using a UV–Vis microplate reader (Synergy HT, BioTek). Results were expressed as mg Trolox/L.

#### 2.5.2 Ferric reducing antioxidant power (FRAP) assay

Ferric reducing antioxidant power (FRAP) was determined according to the method described by Viteri et al. (2022). The FRAP reagent was prepared using 0.3 M acetate buffer (pH 3.6), 10 mM TPTZ in 40 mM HCl, and 20 mM FeCl_3_·6H_2_O. In 96-well microplates, 20 µL of sample was mixed with 180 µL of FRAP reagent, incubated in the dark for 20 min, and the absorbance was measured at 600 nm using a UV–Vis microplate reader (Synergy HT, BioTek). Results were expressed as µM Trolox/L.

#### 2.5.3 ABTS (2,2-azino-bis-3-ethylbenzothiazoline-6-sulphonic acid) assay

The antioxidant activity of the hydrosol was determined using the ABTS assay as described by Viteri et al. (2022). In 96-well microplates, 50 µL of the sample was mixed with 150 µL of ABTS^+^ solution (169 µM) and incubated in the dark for 30 min. Absorbance was recorded at 734 nm using a UV–Vis microplate reader (Synergy HT, BioTek). A Trolox calibration curve (50–200 µM) was employed, and results were expressed as mg Trolox/L.

### 2.6 Determination of total flavonoid content

The total flavonoid content was quantified by complex formation with aluminum chloride, following the method of Chóez-Guaranda et al. (2020). In 96-well plates, 20 µL of the sample was mixed with 200 µL of 10% AlCl_3_ in ethanol and incubated for 30 min at room temperature. Absorbance was measured at 415 nm (Synergy HT reader) using a calibration curve with quercetin (10–100 mg/L). Results were expressed as mg of quercetin equivalents per liter of sample (mg QE/L).

### 2.7 Determination of total polyphenol content

Total phenolic content was quantified using the Folin–Ciocalteu (FC) colorimetric method, following an adapted methodology from Chóez-Guaranda et al. (2020). For the assay, a reaction mixture consisting of 20 µL of sample, 100 µL of diluted Folin–Ciocalteu reagent (1:10, v/v), and 80 µL of 7.5% (w/v) sodium carbonate was prepared. The mixture was incubated for 60 min at room temperature, after which absorbance was measured at 760 nm using a UV–Vis microplate reader (Synergy HT, BioTek). A calibration curve was constructed using gallic acid (10–500 mg/L), and results were expressed as milligrams of gallic acid equivalents per liter of sample (mg GAE/L).

### 2.8 Quantification of polyphenols by high-performance liquid chromatography coupled to a photodiode array detector (HPLC-PDA)

Polyphenol determination was carried out by high-performance liquid chromatography (HPLC) using an Arc HPLC system (Waters Corporation, Milford, MA, United States) equipped with a quaternary pump and a photodiode array detector (PDA). Chromatographic separation was performed on a Zorbax Eclipse XDB-C18 reversed-phase column (4.6 × 150 mm, 5 µm particle size). The chromatographic procedure was based on the method described by Sánchez-Bonet et al. (2021), with modifications according to Gonçalves et al. (2022).

The mobile phase consisted of two eluents: eluent A, HPLC-grade methanol containing 0.2% (v/v) formic acid, and eluent B, ultrapure water containing 0.2% (v/v) formic acid. A gradient elution program was applied as follows: 20% A at the initial time, increased to 32.5% A during the first 15 min, then increased to 70% A at 35 min. Initial conditions were restored at 36 min and maintained until the end of the run at 39 min. The flow rate was maintained at 1.0 mL/min, the injection volume was 10 μL, and the column temperature was set at 30 °C. Prior to analysis, all samples were filtered through 0.45 µm membrane filters. Detection was performed using the PDA detector at 280 nm for, gallic acid, catechin, epicatechin and syringic acid, and at 320 nm for chlorogenic acid, caffeic acid, *p*-coumaric acid, rutin, quercetin, naringenin, kaempferol and apigenin. All analyses were performed in triplicate. Compound identification was carried out by comparing retention times and UV spectra with those of reference standards (Sánchez-Bonet et al., 2021).

### 2.9 Anti-inflammatory activity: inhibition of COX-1 and COX-2 by ELISA (Cayman kit)

The assessment of anti-inflammatory capacity was performed using a competitive ELISA method, based on an enzymatic reaction catalyzed by cyclooxygenases (COX1 and COX2), following the standardized procedure of Cayman Chemical (2023) with methodological adaptations from (Schultz et al., 2021). The extracts were prepared at five concentrations: 450, 250, 100, 50, and 25 μg/mL. For the assay, two reactions were carried out. The first involved generating human prostaglandin H2 (PGH-2) by incubating arachidonic acid with the sample for 2 min at 37 °C. In the second step, the derived prostaglandin (PGF_2_α) was quantified by ELISA in 96-well plates. Detection was performed with Ellman’s reagent, incubating for 90 min at 37 °C and recording absorbance at 412 nm using a UV–VIS microplate reader (Synergy HT, BioTek). A calibration curve of PGF_2_α (3.91–500 μg/mL) was used, and aspirin (COX-1) and celecoxib (COX-2) served as positive controls. Results were expressed as percentage inhibition of prostaglandin (PGF_2_α) (Schultz et al., 2021).

### 2.10 Antibacterial activity

The antimicrobial activity of the *J. osteosperma* (Torr.) Little hydrosol was evaluated against five clinically relevant pathogenic bacteria. The strains used belonged to the CIBE collection and were reactivated in Mueller–Hinton medium.

- CCMCIBE B1049: *Listeria monocytogenes*
- CCMCIBE B1059: *Salmonella entérica*
- CCMCIBE B662: *Staphylococcus saprophyticus*
- CCMCIBE B1036: *Escherichia. Coli*
- CCMCIBE B1263: *Klebsiella variicola*

Antibacterial activity was assessed using the agar diffusion method with 5 mm wells. Plates were prepared with Müller–Hinton medium and inoculated with 100 µL of each bacterial suspension adjusted to 0.5 on the McFarland scale, ensuring uniform distribution across the agar surface (Hossain, 2024; Ramirez and Castaño, 2009). Ampicillin at 100 μg/mL was used as the positive control. The plates were incubated at 37 °C for 24 h. After incubation, the inhibition zones around the wells were examined to assess antimicrobial activity (Ramirez and Castaño, 2009; Chafla Moina and Silva Deley, 2015).

### 2.11 Statistical analysis

In the present study, a representative batch of hydrosol provided by Young Living was analyzed. All experimental determinations were performed from three independent preparations derived from the same batch (n = 3), each analyzed through technical replicates derived from each preparation. Results were expressed as mean ± standard deviation (SD) (Miller and Miller, 2018).

The chemical characterization of the essential oil by GC–MS was carried out using a qualitative–descriptive approach, reporting the relative composition of the identified compounds based on mass spectral comparison and retention indices, in accordance with standardized methodologies for volatile compound analysis (Adams, 2017). For the colorimetric assays (DPPH, ABTS, FRAP, total phenolic content, and total flavonoid content), determinations were performed under the same experimental scheme (n = 3), and results were expressed as mean ± SD. In the HPLC-PDA chromatographic analysis, samples were analyzed from three independent preparations (n = 3), and results were reported as mean ± SD. Compound identification was performed by comparing retention times and UV–Vis spectra with reference standards.

For the evaluation of anti-inflammatory activity, the previously concentrated hydrosol was tested at five concentration levels (25–450 μg/mL), prepared under the same experimental scheme (n = 3). The percentage of COX inhibition was calculated using the Excel template provided by Cayman Chemical. Dose–response analysis was performed by nonlinear regression using a log (inhibitor) vs. response model with variable slope (four-parameter logistic model), implemented in GraphPad Prism version 9.0.0, from which IC_50_ values were estimated (Motulsky and Christopoulos, 2004; Sebaugh, 2011).

Additionally, inferential statistical analyses were performed using the original replicate inhibition values (n = 3) prior to template processing. One-way analysis of variance (ANOVA) was conducted to evaluate the effect of concentration on COX-1 and COX-2 inhibition, followed by Tukey’s *post hoc* test when applicable. Student’s t-test was used to compare COX-1 and COX-2 inhibition at each concentration. All statistical analyses were performed using R software (version 4.3.1), and statistical significance was set at p < 0.05.

Antibacterial activity was evaluated using the agar diffusion method, employing three independent preparations for each bacterial strain (n = 3). Inhibition zone diameters were expressed as mean ± SD, and results were interpreted descriptively, considering the inherent limitations of this method in terms of sensitivity and quantification (Balouiri et al., 2016). Given the exploratory nature of the study, statistical analysis was limited to descriptive statistics and nonlinear regression modeling, without the application of inferential statistical tests.

## 3 Results

### 3.1 Essential oil

The chemical composition of the essential oil obtained from *J. osteosperma* (Torr.) Little wood was analyzed by GC–MS, and the results are presented in Supplementary Table S1. A total of 38 compounds were identified, representing 98.2% of the total oil composition. The essential oil was predominantly composed of oxygenated monoterpenes and sesquiterpenes.

### 3.2 Evaluation of antioxidant activity and total flavonoid and phenolic contents

**FIGURE 1:**
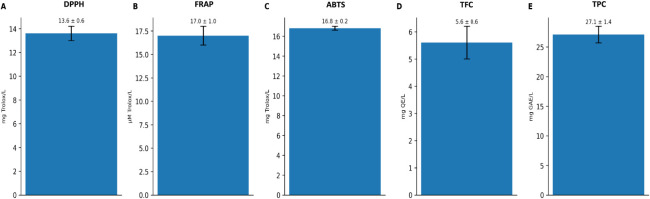
Antioxidant capacity and phytochemical content of *Juniperus osteosperma* (Torr.) Little hydrosol: **(A)** DPPH radical scavenging activity, expressed as mg Trolox/L, **(B)** Ferric reducing antioxidant power (FRAP), expressed as µM Trolox/L, **(C)** ABTS radical cation decolorization assay, expressed as mg Trolox/L, **(D)** Total flavonoid content (TFC), expressed as mg quercetin equivalents per liter (mg QE/L), **(E)** Total phenolic content (TPC), expressed as mg gallic acid equivalents per liter (mg GAE/L). All experiments were performed using unconcentrated hydrosol. Results are expressed as mean ± standard deviation (SD) of three independent preparations (n = 3).

The antioxidant activity of *J. osteosperma* (Torr.) Little hydrosol was evaluated using three complementary spectrophotometric assays: DPPH, ABTS, and FRAP, while total polyphenol and flavonoid contents were determined using the TPC and TFC assays, respectively (Figure 1). Values are expressed as mean (n = 3) ± standard deviation (SD).

The hydrosol exhibited antioxidant capacity in all three assays, with values of 17 ± 1.0 µM Trolox/L for FRAP, 16.8 ± 0.2 mg Trolox/L for ABTS, and 13.6 ± 0.6 mg Trolox/L for DPPH. The highest level of activity was observed in the FRAP assay, followed by ABTS and DPPH, indicating a preference for electron transfer (SET) over hydrogen atom transfer (HAT) mechanisms. Total phenolic content (TPC) was 27.1 ± 1.4 mg GAE/L, whereas total flavonoid content (TFC) was 5.6 ± 0.6 mg QE/L.

### 3.3 Phenolic compounds identification by HPLC

Phytochemical characterization of the *Juniperus osteosperma* (Torr.) Little hydrosol by high-performance liquid chromatography (HPLC) enabled the evaluation of a panel of 13 phenolic compounds and flavonoids of pharmacological interest, including flavanones, flavonols, flavones, and phenolic acids. However, only one compound was detected and quantified, while the remaining 12 compounds were not detected at quantifiable levels under the analytical conditions employed (Table 1). Representative chromatograms of the hydrosol (replicates) and standard compounds recorded at 280 nm and 320 nm are provided in the Supplementary Material S2.

**TABLE 1:** Bioactive compounds identified in *Juniperus osteosperma* (Torr.) Little hydrosol, by HPLC, and chromatograms of Naringenin.

Compound	*Juniperus osteosperma* (torr.) little hydrosol (mg/L ± de)	​
Apigenin	NC	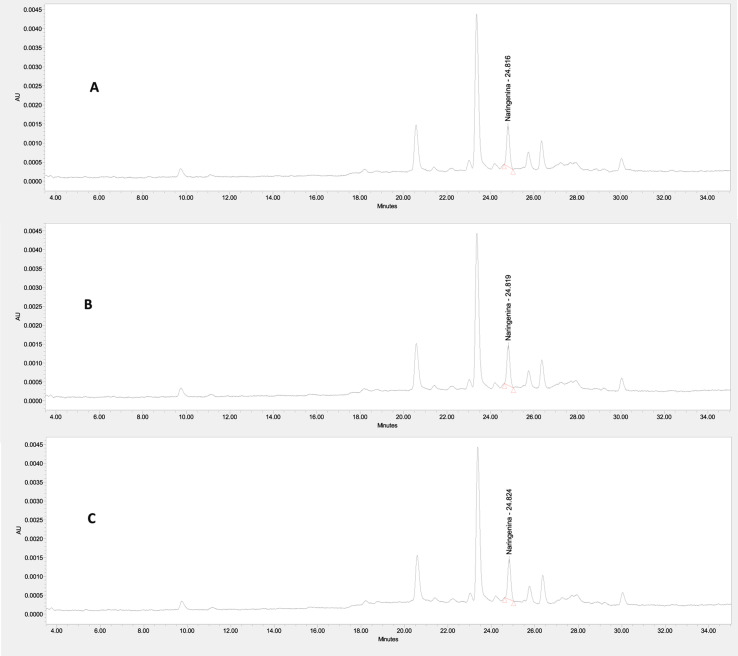
Caffeine	NC	
Catechin	NC	
Epicatechin	NC	
Kaempferol	NC	
Caffeic acid	NC	
Quercetin	NC	
Rutin	NC	
p-coumaric acid	NC	
Syringic acid	NC	
Chlorogenic acid	NC	
Naringenin	2.98 ± 0.02	

The results are expressed as mean values (n = 3) accompanied by the corresponding standard deviation. Outlier values were excluded from the analysis. The percentage of peak area represents the relative abundance of each detected compound. The abbreviation NC, refers to compounds not found in the quantification. The experiments shown were performed using the unconcentrated hydrosol.

The results are expressed as mean values (n = 3) accompanied by the corresponding standard deviation. Outlier values were excluded from the analysis. The percentage of peak area represents the relative abundance of each detected compound. The abbreviation NC refers to compounds not found in the quantification. The experiments shown were performed using the unconcentrated hydrosol. The quantifiable flavonoid was naringenin, at 2.98 ± 0.02 mg/L, whereas quercetin, apigenin, rutin, catechin, gallic acid, and chlorogenic acid were not detected (ND). This result corresponds exclusively to the chemical profile of the hydrosol under the analytical conditions employed.

### 3.4 Anti-inflammatory activity: inhibition of COX-1 and COX-2 by ELISA

Percentage inhibition and IC_50_ values were calculated using the Excel template provided by Cayman Chemical, and results were expressed as mean (n = 3) ± standard deviation (SD) (Table 2).

**TABLE 2:** Inhibition and IC_50_ of COX-1 and COX-2 by ELISA.

Sample	Concentration	Percentage of inhibition (%)		Positive control (%)		p-value
	(µg PGF2α/mL ES)	COX-1	COX-2	Aspirina COX-1	Celecoxib COX-2	(COX-1 vs. COX-2)
*Juniperus osteosperma* (Torr.) little hydrosol	450	60.4 ± 1.3	59.7 ± 1.9	77.7. ± 2.1	69.7 ± 1.7	0.626
	250	51.8 ± 2.1	50.1 ± 3.0	71.5 ± 0.56	64.3 ± 0.9	0.466
	100	46.9 ± 2.9	41.6 ± 6.3	69.8 ± 11.9	62.3 ± 2.4	0.256
	50	34.5 ± 1.0	30.3 ± 7.0	65.6 ± 13.6	59.8 ± 2.4	0.362
	25	28.5 ± 5.8	22.1 ± 2.5	61.1 ± 5.8	56.9 ± 1.8	0.154
IC_50_ (µg PGF2α/mL ES)		195 ± 0.2	249 ± 2.3	20.5 ± 1.2	22 ± 3.2	-
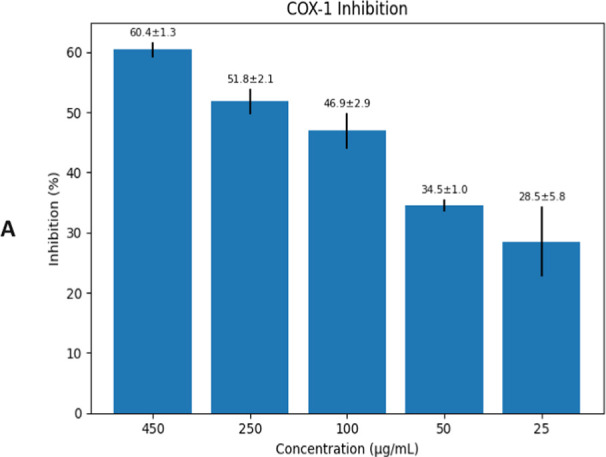				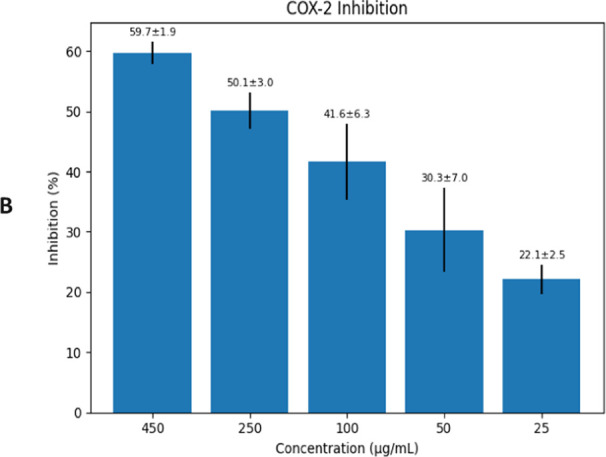		

Values are expressed as mean ± standard deviation (n = 3). Variability was additionally assessed using standard error of the mean (SEM) and coefficient of variation (%CV). p-values correspond to Student’s t-test comparing COX-1, and COX-2, inhibition at each concentration.

The *Juniperus osteosperma* (Torr.) Little hydrosol exhibited dose-dependent inhibition of cyclooxygenase-1 (COX-1) and cyclooxygenase-2 (COX-2). At a concentration of 450 μg/mL, inhibition reached 60.4% ± 1.3% for COX-1 and 59.7% ± 1.9% for COX-2, whereas at 25 μg/mL, inhibition values of 28.5% ± 5.8% and 22.1% ± 2.5% were observed, respectively. Statistical analysis confirmed a significant concentration-dependent effect for both enzymes (p < 0.001, one-way ANOVA), demonstrating a clear dose–response relationship. Importantly, no statistically significant differences were observed between COX-1 and COX-2 inhibition at any of the tested concentrations (Student’s t-test, p > 0.05 in all cases), indicating a comparable inhibitory response between both isoforms under the experimental conditions evaluated (Supplementary Figue S4). R script used for statistical analysis of COX inhibition data.

The estimated IC_50_ values for the hydrosol were 195 ± 0.2 µg PGF2α/mL ES for COX-1 and 249 ± 2.3 µg PGF2α/mL ES for COX-2, indicating a moderate magnitude of *in vitro* inhibitory activity. The reference drugs aspirin and celecoxib were included solely as positive controls for the ELISA assay, yielding IC_50_ values of 20.5 ± 1.2 µg PGF2α/mL ES for aspirin (COX-1) and 22.0 ± 3.2 µg PGF2α/mL ES for celecoxib (COX-2), thereby confirming the method’s sensitivity and performance.

### 3.5 Antibacterial activity

In the agar diffusion assay, *Juniperus osteosperma* (Torr.) Little hydrosol did not produce detectable inhibition zones against any of the five tested strains (*Listeria monocytogenes, Salmonella enterica, Staphylococcus saprophyticus, Escherichia coli, and Klebsiella variicola*). In contrast, the positive control (ampicillin, 100 μg/mL) produced well-defined inhibition zones for all strains, confirming bacterial viability and the proper execution of the assay (Table 3; Supplementary Figure S3).

**TABLE 3:** Antibacterial Activity *Juniperus osteosperma* (Torr.) Little hydrosol.

Sample	Concentration	*L Monocytogenes*	*S. Enterica*	*S. Saprophyticus*	*E. Coli*	*K Variicola*
*Juniperus osteosperma* (torr.) little hydrosol	100%	-	-	-	-	-
		-	-	-	-	-
		-	-	-	-	-
Ampicillin	100 ug/mL	1.2 mm	1.8 mm	0.9 mm	0.5 mm	0.7 mm

Pathogen	Replicate 1	Replicate 2	Replicate 3	Control (−)	Control (+)
*Listeria monocytogenes*	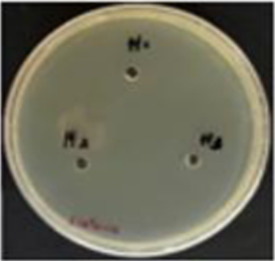	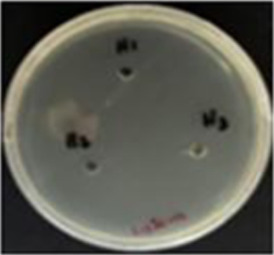	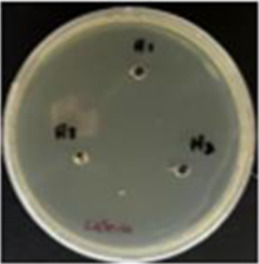	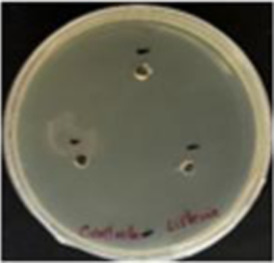	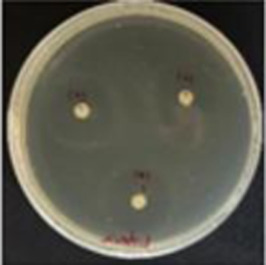
*Salmonella entérica*	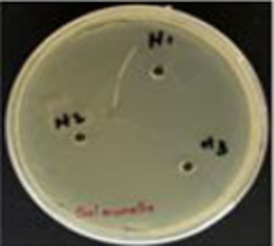	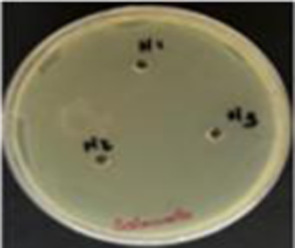	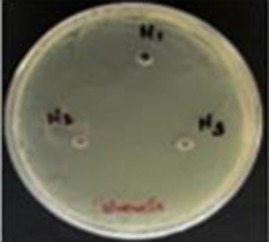	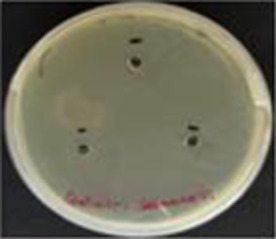	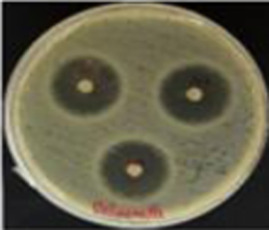
*Staphylococcus Saprophyticus*	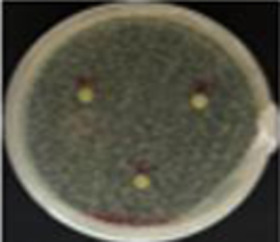	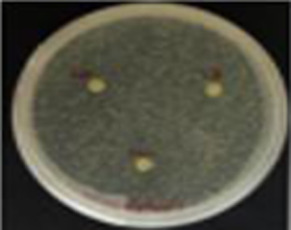	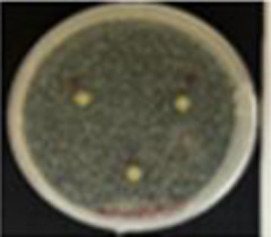	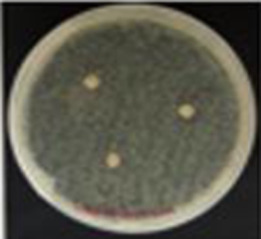	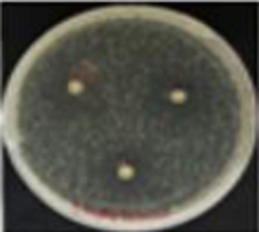
*Eschrerichia. Coli*	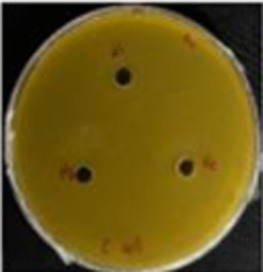	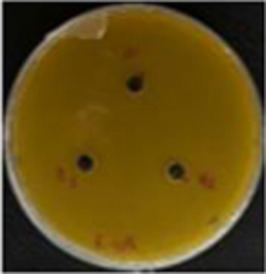	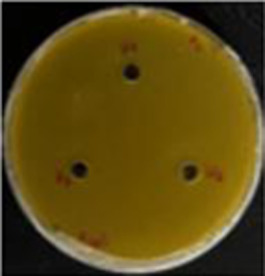	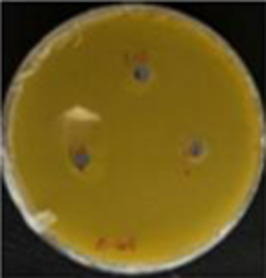	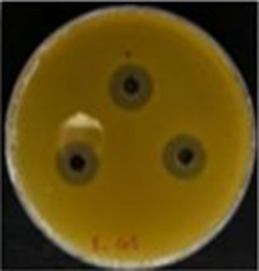
*Klebsiella variicola*	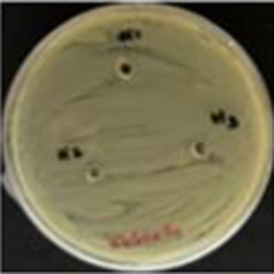	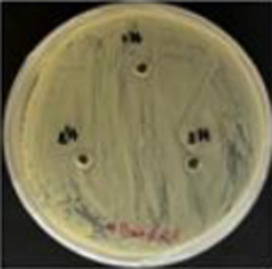	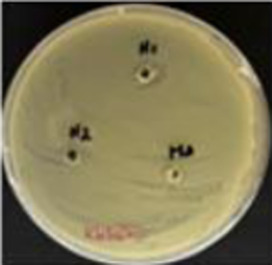	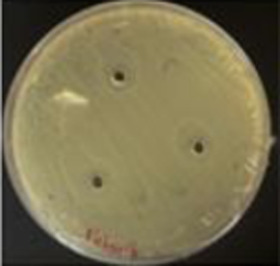	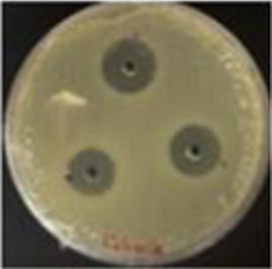

Results are expressed as mean values (n = 3) accompanied by the corresponding standard deviation. The experiments shown were performed using the unconcentrated hydrosol.

## 4 Discussion

This study offers an initial experimental approach to the characterization of *Juniperus osteosperma* (Torr.) Little hydrosol, with emphasis on its phytochemical profile, antioxidant activity, and capacity to modulate the inflammatory enzymes COX-1 and COX-2.

The essential oil of *J. osteosperma* (Torr.) Little exhibited a phytochemical profile predominantly composed of oxygenated monoterpenes and sesquiterpenes, with cis-thujopsene (14.3%), bornyl acetate (10.6%), camphor (8.3%), and cedrol (8.3%) as the major constituents, along with lower proportions of α-cedrene, terpineol, and borneol. This composition differs from that reported for other species within the genus, such as *Juniperus communis* and *Juniperus chinensis*, which are typically rich in α-pinene, sabinene, and limonene. Although some species, including Juniperus thurifera and *Juniperus foetidissima*, share metabolites such as cedrol and widdrol, their relative abundances differ from those observed in *J. osteosperma* (Torr.) Little. These variations may be attributed not only to taxonomic differences but also to ecological and methodological factors, including edaphoclimatic conditions, water stress, altitude, harvesting season, and the plant organ used. Additionally, extraction parameters such as distillation method, duration, and temperature, as well as the analytical techniques employed for compound identification and quantification, can significantly influence the resulting chemical profiles (Höferl et al., 2014; Tsao et al., 2025; Gonçalves et al., 2022).

The antioxidant activity of *J. osteosperma* (Torr.) Little hydrosol was 17 ± 1 µM Trolox/L in the FRAP assay, 16.8 ± 0.2 mg Trolox/L in the ABTS assay, and 13.6 ± 0.6 mg Trolox/L in the DPPH assay, indicating a detectable reducing capacity. The observed pattern (FRAP > ABTS > DPPH) is consistent with the underlying principles of each method: electron-transfer-based assays tend to be more sensitive to hydrosoluble and oxygenated compounds, whereas the DPPH radical exhibits limited responsiveness in aqueous matrices (Suhag et al., 2025). These values are lower than those reported for essential oils and organic extracts of the genus Juniperus. For instance, Raina et al. (2019) reported higher antioxidant activity for aqueous and ethanolic extracts of *J. communis* fruits at concentrations of 20, 40, and 60 μg/mL. Similarly, Fejér et al. (2022) reported IC_50_ values against DPPH and ABTS in the range of 20–80 μg/mL for essential oils from various Juniperus species. These differences can be attributed to the aqueous and highly diluted nature of hydrosols, which contain only the hydrosoluble fraction of bioactive metabolites, as well as to interspecific variability, environmental growth conditions, and extraction methods (Tanjga et al., 2022; Semerdjieva et al., 2021; Gonçalves et al., 2022).

The total phenolic content (27.1 ± 1.4 mg GAE/L) and total flavonoid content (5.6 ± 0.6 mg QE/L) determined in *J. osteosperma* (Torr.) Little hydrosol are low in absolute terms, which is consistent with the highly diluted nature of hydrosols. Previous studies on hydrosols from the genus Juniperus have reported low but detectable phenolic concentrations within ranges comparable to those observed in the present study, and these have been associated with residual biological activity rather than high-potency effects (Aćimović et al., 2020). In contrast, organic extracts from the same genus exhibit substantially higher concentrations; for example, Fejér et al. (2022) reported total phenolic contents ranging from 20–90 mg GAE/g dry extract and total flavonoids between 2–25 mg QE/g in methanolic and ethanolic extracts of *J. communis*, while Miceli et al. (2011) reported a total phenolic content of 48.06 ± 0.99 mg GAE/g dry extract for Juniperus drupacea. These quantitative differences reflect the greater extraction efficiency of organic solvents and the expression of results on a dry-weight basis, in contrast to hydrosols, whose values are reported per volume of aqueous distillate.

However, these colorimetric results must be interpreted with caution. Both the Folin–Ciocalteu and aluminum chloride (AlCl_3_) assays were originally developed and are primarily validated for organic or hydroalcoholic plant extracts rather than highly diluted aqueous distillates such as hydrosols. In this context, the Folin–Ciocalteu assay should not be considered a specific measurement of true phenolic concentration, but rather an estimate of the overall reducing capacity of Folin-reactive substances, since non-phenolic reducing compounds may also contribute to the signal. Similarly, the aluminum chloride (AlCl_3_) method provides only an operational estimate of flavonoid-like reactivity and may be influenced by compound structure, low analyte abundance, and non-specific interactions in aqueous matrices. Therefore, the TPC and TFC values reported here should be regarded as preliminary indicators of reducing constituents and flavonoid-like compounds rather than definitive quantitative evidence of phenolic and flavonoid abundance in the hydrosol. This limitation is particularly relevant in hydrosols, where analyte concentrations are low and matrix effects may have a proportionally greater impact on absorbance-based measurements. Methods (Tanjga et al., 2022; Semerdjieva et al., 2021; Gonçalves et al., 2022).

The phenolic profile of *J. osteosperma* (Torr.) Little hydrosol and the influence of the extraction method on its phytochemical complexity were evaluated by HPLC, under which conditions a single quantifiable peak with flavonoid-like behavior was tentatively assigned as naringenin. This finding should also be interpreted conservatively. From a physicochemical perspective, most flavonoids are non-volatile compounds and would not be expected to transfer efficiently during steam distillation or hydrodistillation. Accordingly, the apparent detection of a flavonoid in the hydrosol may reflect a trace-level transfer phenomenon, but it may also arise from co-elution, matrix-related interference, contamination during distillation or sample handling, or limitations of UV-based compound assignment in a highly diluted aqueous matrix. In this sense, the observed signal should not be interpreted as unequivocal evidence of substantial flavonoid transfer into the hydrosol. Nevertheless, this finding remains consistent with the chemically constrained phytochemical profile commonly associated with hydrosols. According to Aćimović et al. (2020), these distillation by-products retain only a limited fraction of phenolic constituents, reflecting the selective partitioning behavior characteristic of steam distillation. The predominance of volatile and water-soluble components, together with the limited transfer of less volatile phenolics, likely explains the reduced phytochemical complexity observed in the present analysis. At the same time, because the concentration of genuine phenolic constituents in hydrosols is expected to be very low, any chromatographic signal detected near the method’s sensitivity limit must be critically evaluated (Fejér et al., 2022).

In contrast, studies using organic solvent extracts of Juniperus species consistently report a broader and more complex phenolic composition. Notably, Miceli et al. employed HPLC-DAD-ESI-MS to characterize *Juniperus drupacea* extracts, which were found to be rich in phenolic acids and flavonoids, with amentoflavone identified as a dominant constituent. Similarly, Kalam (2020) identified several flavonoids, including quercetin, rutin, and amentoflavone, in organic extracts of *J. communis*. It is important to note, however, that these studies used organic extraction methods and more sensitive analytical techniques, including mass spectrometric detection, whereas the present analysis relied on hydrosol extraction and HPLC with UV/PDA detection. Therefore, while the overall impact of extraction methodology on detectable phenolic content is evident, some differences in the observed phytochemical spectrum may also arise from variations in analytical sensitivity, extraction selectivity, and matrix complexity. These considerations are particularly important when interpreting the tentative identification of naringenin in *J. osteosperma* (Torr.) Little hydrosol. Given that most flavonoids are poorly compatible with transfer via steam distillation, the presence of a naringenin-like peak in the chromatogram should be regarded as a provisional analytical observation rather than definitive structural confirmation. Accordingly, the tentative identification of naringenin should be considered valid only under the specific chromatographic conditions applied and for the analyzed hydrosol batch. Definitive confirmation would require more advanced analytical approaches, such as LC–MS/MS, standard addition strategies, or co-injection with authentic standards.

Regarding anti-inflammatory activity, the hydrosol demonstrated a clear dose-dependent inhibition of both cyclooxygenase isoforms. Inhibition values ranged from 28.5% to 60.4% for COX-1 and 22.1%–59.7% for COX-2 as the concentration increased from 25 to 450 µg PGF2α/mL ES, with inhibition approaching 60% only at the highest concentration tested. This trend was statistically supported by a significant effect of concentration on enzyme inhibition (p < 0.001, one-way ANOVA). In contrast, no statistically significant differences were observed between COX-1 and COX-2 inhibition at equivalent concentrations (p > 0.05, Student’s t-test), suggesting a comparable inhibitory response toward both isoforms. The calculated IC_50_ values (195 ± 0.2 μg/mL for COX-1 and 249 ± 2.3 μg/mL for COX-2) indicate a moderate inhibitory effect of the hydrosol under the experimental conditions. In a pharmacological context, previous *in vivo* studies have shown that plasma concentrations of naringenin, as a pure compound, typically peak below 10 μg/mL following oral administration in humans. Furthermore, effective anti-inflammatory concentrations in cell-based and animal studies are generally observed in the low to mid-micromolar range, corresponding to approximately 1–10 μg/mL. The concentrations required for 50% inhibition in the present study are therefore substantially higher than those likely to be achieved in physiological or clinical settings, suggesting limited bioactivity at attainable topical or systemic levels. Nevertheless, uncertainties persist due to possible differences in bioavailability and matrix effects in hydrosols, as well as the potential for additive or synergistic effects among multiple minor constituents.

Compared with aqueous or hydroalcoholic extracts of *Juniperus* species, the magnitude of inhibition observed in this study is lower, consistent with the reduced phenolic complexity typically associated with hydrosols. For example, Kachmar et al. (2024) reported significant downregulation of COX expression in cellular models treated with aqueous extracts of *Juniperus*. Similarly, Raina et al. (2019) demonstrated that aqueous extracts of *J. communis* inhibited prostaglandin biosynthesis by approximately 55%, while hydroalcoholic preparations exhibited comparable or enhanced activity. *In vivo*, Gonçalves et al. (2022) observed up to a 79% reduction in carrageenan-induced edema following treatment with hydroethanolic extracts of *J. communis*, accompanied by antihistaminic and antiserotonergic effects, findings further corroborated by previous studies (Gonçalves et al., 2022; Zhao et al., 2018; Raina et al., 2019; Kachmar et al., 2024).

In contrast, *J. osteosperma* (Torr.) Little hydrosol did not produce inhibition halos in the agar diffusion assay. This result should not be regarded as definitive evidence of the absence of antibacterial activity, since agar diffusion methods have well-documented limitations when applied to highly hydrophilic matrices such as hydrosols. The reduced capacity of these matrices to diffuse efficiently through solid media can substantially underestimate biological activity, particularly for compounds present at low concentrations, as discussed by Karaman et al. (2003). The absence of observable inhibition zones aligns with the findings of Mediavilla et al. (2023), who reported minimal or no biocidal effects for aqueous fractions and hydrosol of *J. communis*, in contrast to essential oils from the same genus, which exhibited moderate to strong antimicrobial activity. Similarly, Karaman et al. (2003) demonstrated that aqueous extracts of *Juniperus* species showed limited antimicrobial activity against a range of microorganisms, whereas organic extracts were more active. These findings are particularly relevant given the growing recognition of hydrosols as environmentally friendly, sustainable alternatives to conventional antimicrobials; increased research into their mechanisms and efficacy could inform future development of green antimicrobial solutions. Within this context, the present results likely reflect methodological constraints inherent to the agar diffusion assay rather than a genuine lack of antibacterial potential. Future investigations employing liquid-phase methodologies, such as broth microdilution assays for determining minimum inhibitory concentration (MIC), would provide a more sensitive and quantitative assessment of the antibacterial activity of the hydrolate.

Despite the methodological constraints inherent to the analytical approaches employed, the present study demonstrates that *J. osteosperma* (Torr.) Little hydrosol retains water-soluble metabolites associated with measurable *in vitro* antioxidant and anti-inflammatory activities. Although the magnitude of these effects is moderate, their detection supports the view that hydrosols, traditionally regarded as low-value distillation by-products, may preserve biologically relevant constituents within the aqueous fraction. At the same time, the present findings should not be overinterpreted as definitive proof of substantial flavonoid content in the hydrosol, since both the chromatographic and colorimetric evidence remain method-dependent and susceptible to matrix interference. Rather, the data support the more conservative conclusion that *J. osteosperma* (Torr.) Little hydrosol contains low-abundance redox-active and bioactive water-soluble constituents that warrant further characterization.

These findings expand current knowledge of the chemical composition and bioactivity of hydrosols derived from the *Juniperus* genus and establish a scientific framework for future investigations focused on comprehensive phytochemical characterization and functional evaluation of this natural matrix. Integrative analytical strategies that combine high-resolution metabolomic profiling with mechanistic bioassays will be essential to fully elucidate the therapeutic and biotechnological potential of *J. osteosperma* (Torr.) Little.

## 5 Conclusion

This study provides an initial experimental characterization of *J. osteosperma* (Torr.) Little hydrosol, a distillation by-product traditionally regarded as low-value, demonstrating that it retains water-soluble constituents associated with measurable *in vitro* antioxidant and anti-inflammatory activities. Although the total phenolic content was low compared to organic extracts and essential oils, *J. osteosperma* (Torr.) Little hydrosol exhibited dose-dependent inhibition of COX-1 and COX-2, reaching only moderate effects at the highest concentrations tested. These findings suggest that its bioactivity is likely constrained by the low abundance of active constituents in the aqueous matrix.

HPLC–PDA analysis enabled the tentative detection of a flavonoid-like signal consistent with naringenin; however, this observation must be interpreted with caution. Given the non-volatile nature of flavonoids and the analytical limitations associated with highly diluted aqueous matrices, this signal may reflect trace-level presence, co-elution, or matrix-related interference rather than definitive compound identification. Importantly, the flavonoid signal tentatively assigned as naringenin remains unconfirmed and requires validation by mass spectrometric techniques (e.g., LC–MS/MS) before definitive identification can be established. Similarly, the absence of detectable antibacterial activity in the agar diffusion assay is more plausibly attributed to methodological limitations inherent to diffusion-based techniques when applied to hydrophilic systems, rather than conclusive evidence of biological inactivity.

Taken together, these results highlight both the potential and the limitations of *J. osteosperma* (Torr.) Little hydrosol as a complex but low-concentration matrix containing bioactive, water-compatible constituents. Additionally, no statistically significant differences were observed between COX-1 and COX-2 inhibition (p > 0.05), indicating a comparable inhibitory response between both isoforms under the experimental conditions evaluated. Future studies employing high-sensitivity analytical platforms, such as LC–MS/MS, along with validated cellular and toxicological models, will be essential to confirm compound identity, clarify mechanisms of action, and establish the safety and functional relevance of *J. osteosperma* (Torr.) Little hydrosol. Such efforts will be critical to determine its realistic potential for application in dermocosmetic or other biotechnological contexts, particularly within the framework of sustainable utilization of distillation by-products.

## Data Availability

The original contributions presented in the study are included in the article/Supplementary Material, further inquiries can be directed to the corresponding author.
