# Supplementary material for: Antioxidant, anti-inflammatory, and phytochemical properties of Juniperus osteosperma (Torr.) little hydrosol: an in vitro evaluation
Source: Front Pharmacol. 2026 Jun 24;17:1774954. doi: 10.3389/fphar.2026.1774954 (PMC13343228; doi:10.3389/fphar.2026.1774954)
Supplement: Supplementary file 1 [file Supplementaryfile1.pdf]

Supplementary Table S1. GC–MS chemical composition of *Juniperus osteosperma* (Torr.) Little essential oil.

KI	Compound	*Juniperus osteosperma* (Torr.) Little (area %)
921	tricyclene	1.4
932	α-pinene	19.0
946	camphene	0.8
969	sabinene	3.7
974	β-pinene	0.1
988	myrcene	0.7
1008	δ-3-carene	0.3
1014	α-terpinene	0.6
1020	p-cymene	2.3
1024	limonene	3.9
1044	β-ocimene	0.3
1054	γ-terpinene	1.1
1086	terpinolene	0.7
1122	α-campholenal	0.3
1141	camphor	8.3
1145	camphene hydrate	0.3
1165	borneol	2.1
1174	terpinen-4-ol	2.7
1186	α-terpineol	0.5
1194	myrtenol	0.4
1204	verbenone	0.5
1215	trans-carveol	0.3
1241	carvacrol, methyl ether	0.8
1284	bornyl acetate	10.6
1410	α-cedrene	3.6
1419	β-cedrene	1.0
1429	cis-thujopsene	14.3
1452	α-humulene	0.5
1474	acoradiene <10-epi-β->	0.4
1476	β-chamigrene	0.3
1504	cuparene	1.4
1522	δ-cadinene	1.9
1548	elemol	1.2
1599	widdrol	1.3
1600	cedrol	8.3
1636	β-acorenol	1.0
1649	β-eudesmol	0.8
1688	cedren-13-ol	0.5
	**Total**	**98.2**

Kovats retention index calculated by linear interpolation relative to a homologous series of n-alkanes on a DB-5 column, following the methodology described by Adams (2017).

Supplementary S2. Representative HPLC-PDA chromatograms of *Juniperus osteosperma* (Torr.) Little hydrosol obtained from three independent analytical replicates (A, B, C, D, E)

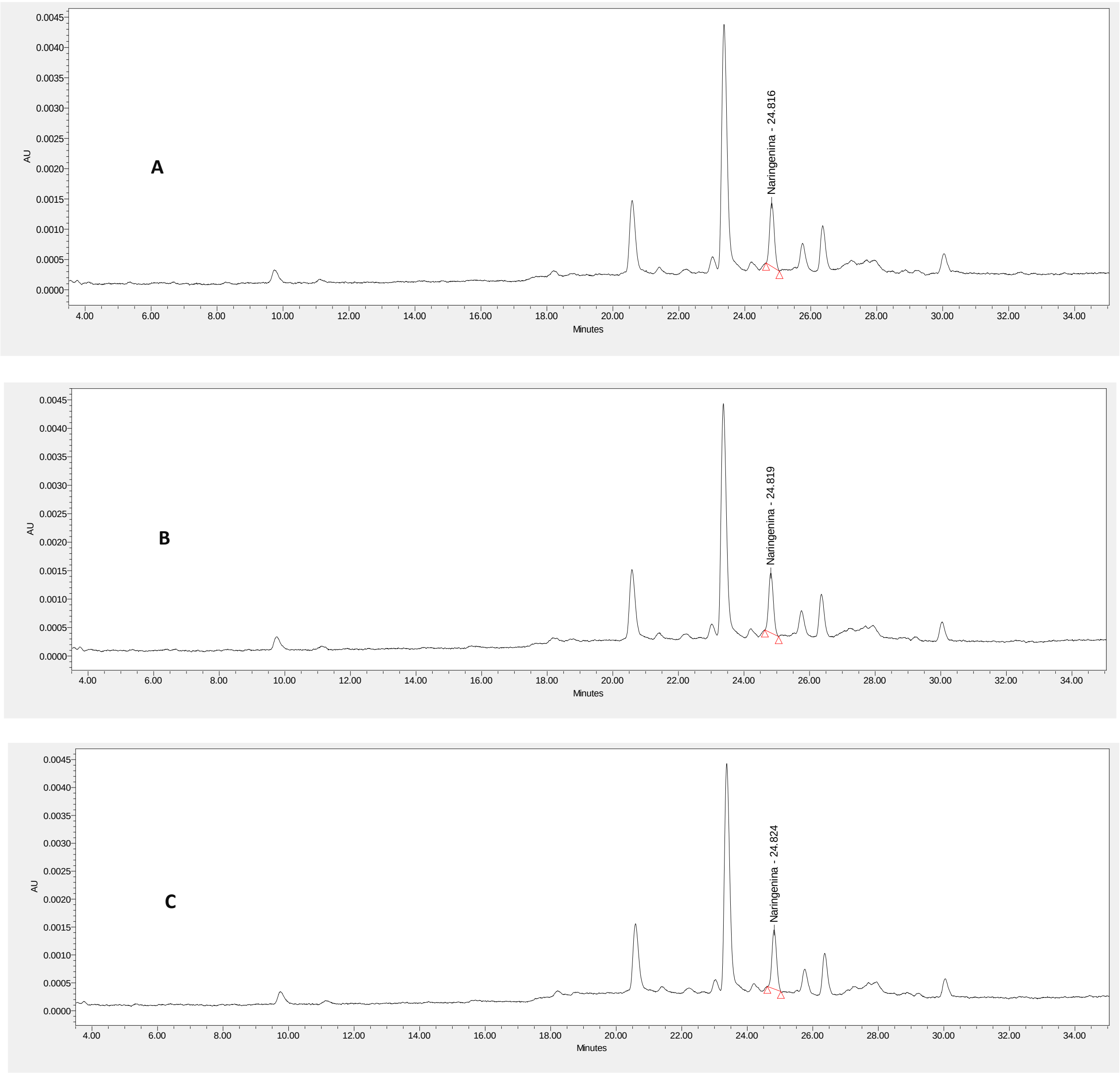

Representative chromatograms of the *Juniperus osteosperma* (Torr.) Little (A–C) corresponding to three independent replicates (n = 3), showing consistent chromatographic profiles.

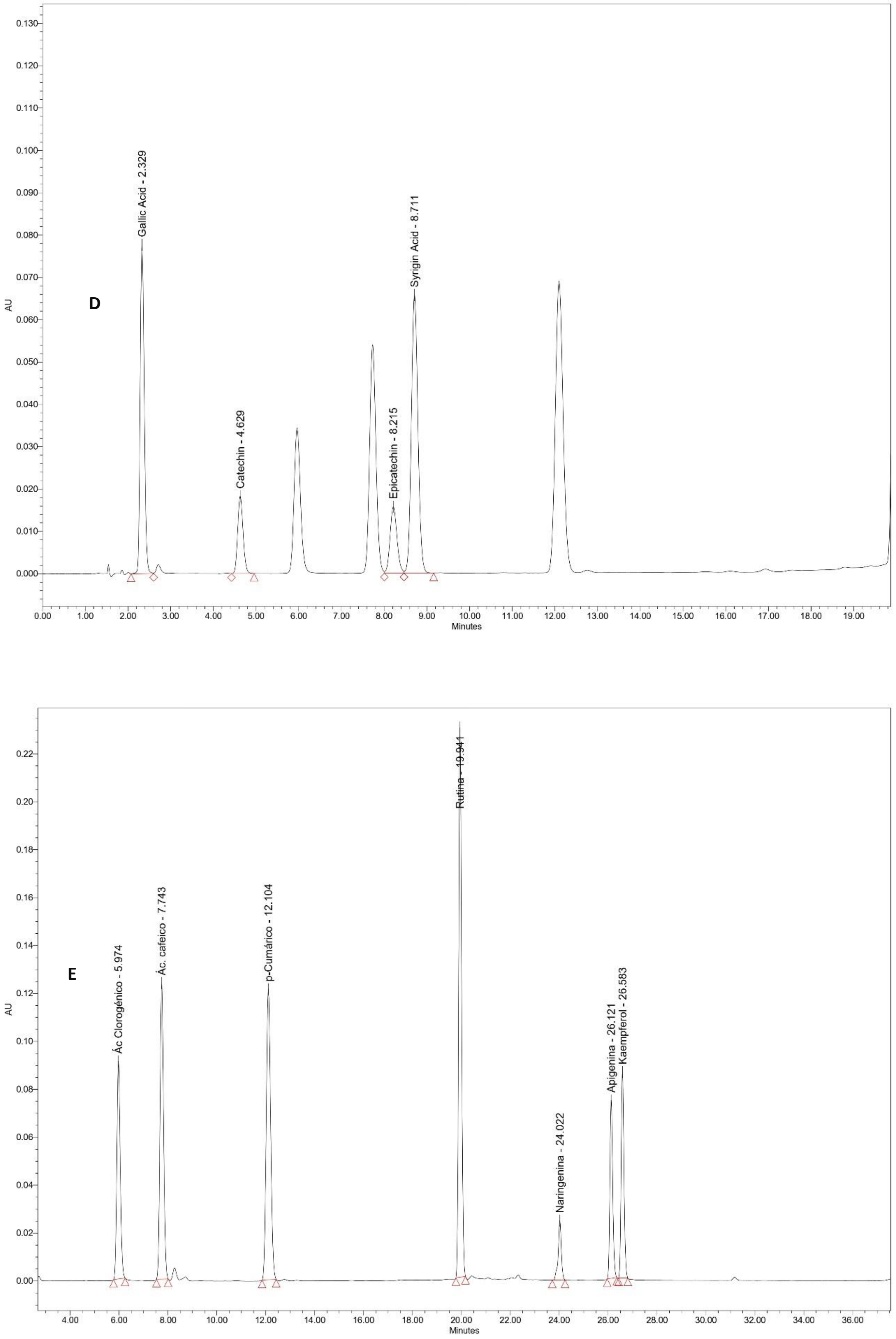

Representative HPLC chromatograms of *Juniperus osteosperma* (Torr.) Little hydrosol and phenolic standards. Panels (A–C) correspond to independent hydrosol replicates (a–c), while panels (D) and (E) show chromatograms of standard compounds recorded at 280 nm and 320 nm, respectively.

Supplementary S3. Dynamics of microbial activity in *Juniperus osteosperma* (Torr.) Little hydrosol

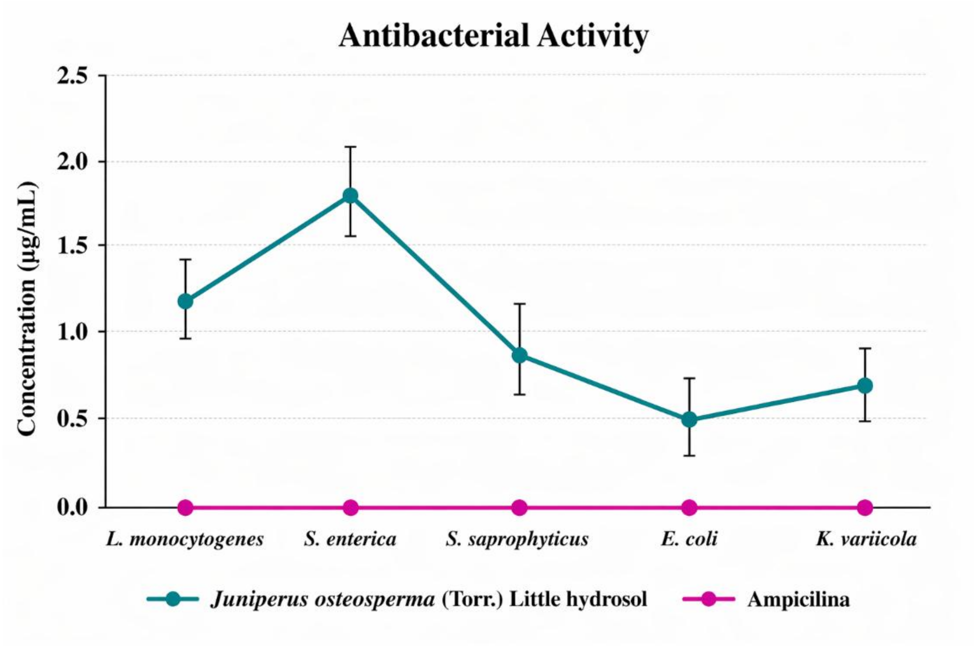

Antibacterial activity of *Juniperus osteosperma* (Torr.) Little hydrosol against selected bacterial strains. Low inhibitory effects were observed across all microorganisms, with slight variation among species. Values are expressed as mean ± SD (n = 3).

Supplementary S4. Assessment of ANOVA assumptions for COX inhibition data

Model	COX-1 (p-value)	COX-2 (p-value)
Normality (Shapiro–Wilk)	0.439	0.291
Homogeneity (Levene)	0.272	0.444
Independence (experimental design)	N/A	N/A

Assessment of the assumptions underlying the analysis of variance (ANOVA) applied to COX inhibition data. Normality of residuals was evaluated using the Shapiro–Wilk test, and homogeneity of variances was assessed using Levene's test. Independence was ensured by the experimental design, as the analysis was performed using three independent preparations (n = 3). No violations of ANOVA assumptions were detected ($p > 0.05$).
